# Crystal structure of caesium tetra­methyl­dithio­imidodiphosphinate

**DOI:** 10.1107/S2056989021009798

**Published:** 2021-09-30

**Authors:** Marcela López-Cardoso, Hugo Tlahuext, Vojtech Jancik, Raymundo Cea-Olivares

**Affiliations:** a Centro de Investigaciónes Químicas, Universidad Autónoma del Estado de Morelos, Av. Universidad No. 1001, Col. Chamilpa, CP 62209, Cuernavaca, Mor., Mexico; b Centro Conjunto de Investigación en Química Sustentable UAEM-UNAM, Carretera Toluca-Atlacomulco Km. 14.5, Toluca, 50200, Estado de México, Mexico; c Instituto de Química, Universidad Nacional Autónoma de México, Circuito Exterior, Ciudad Universitaria, México, 10810, Ciudad de México, Mexico

**Keywords:** crystal structure, caesium, tetra­methyl­dithio­imidodiphosphinate anion, two-dimensional polymer structure

## Abstract

In the title crystal, the salt [CsMe_2_P(S)NP(S)Me_2_] is self-assembled as an undulating supra­molecular two-dimensional polymeric structure, poly[(μ_4_-tetra­methyl­dithio­imidodiphosphinato)caesium], [Cs(C_4_H_12_NP_2_S_2_)]_*n*_, which is parallel to the *bc* plane. The Cs cations are hexa­coordinated, being chelated by two thio­imidophosphinate groups and two sulfur atoms from neighboring ligands. The anions are linked to the Cs cations by Cs⋯S and Cs⋯N electrostatic inter­actions.

## Abstract 2

A supra­molecular two-dimensional polymer structure of caesium tetra­methyl­dithio­imidodiphosphinate is reported.

## Chemical context   

Dichalcogenoimidodiphosphinate anions [*R*_2_P(*E*)NP(*E*)*R*_2_]^−^ (*E* = O, S, Se, Te) are versatile complexing reagents with a strong tendency to form inorganic (carbon-free) chelate rings (Haiduc & Silaghi-Dumitrescu, 1986 ▸; Cea-Olivares & Muñoz, 1993 ▸; Hernández-Arganis *et al.*, 2004 ▸; Slawin *et al.*, 1994 ▸). The monoanionic ligands have been investigated as ligands for both main-group elements (Silvestru & Drake, 2001 ▸; Woollins, 1996 ▸) and transition metals (Rudler *et al.*, 1997 ▸). The widespread inter­est in dichalcogenoimidodiphosphinates stems from their potential uses as lanthanide shift reagents (Rudler *et al.*, 1997 ▸), industrial catalysts (Leung *et al.*, 2000 ▸; Yamazaki *et al.*, 2020 ▸), luminescent materials (Ma *et al.*, 2019 ▸) as well as in metal extraction processes (du Preez *et al.*, 1992 ▸). As part of our ongoing research on dichalcogenoimidodiphosphinate anions, we report herein the synthesis and crystallographic study of the title compound (**I**).
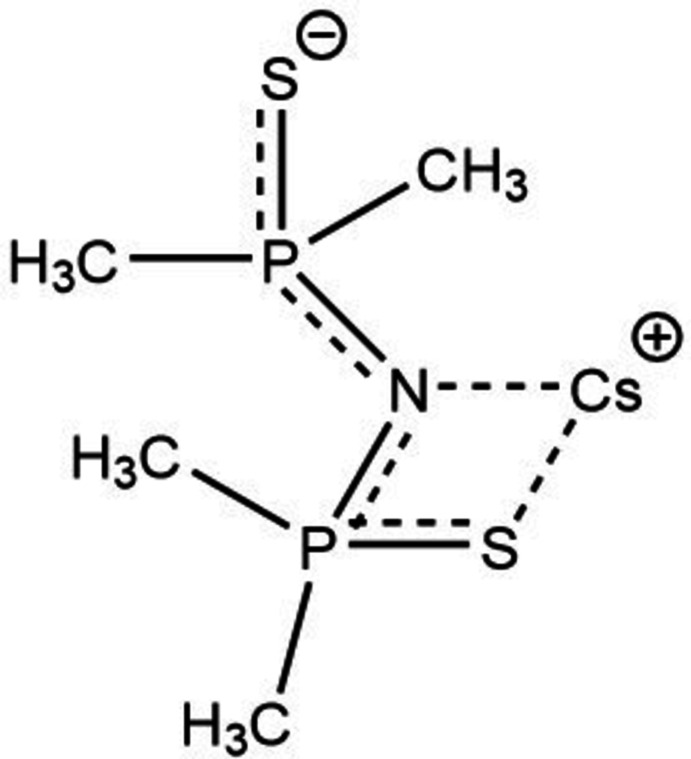


## Structural commentary   

**Figure 1:**
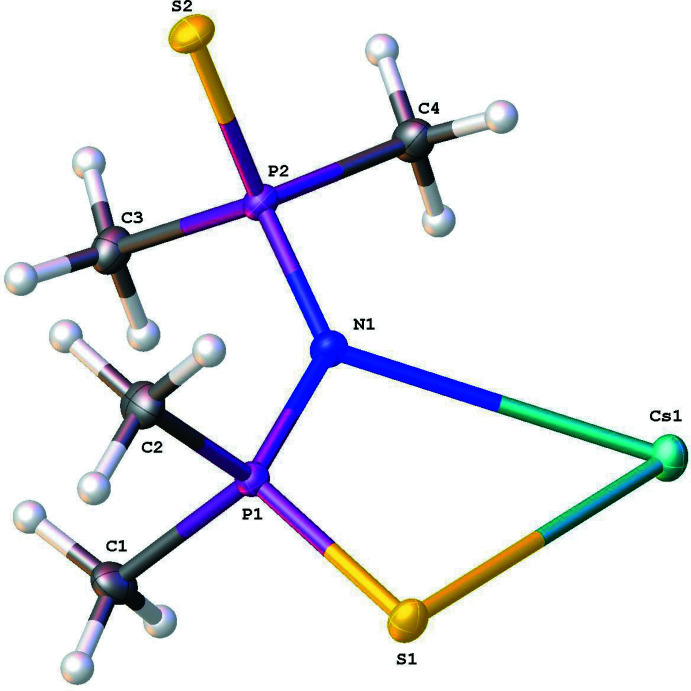
The asymmetric unit of the title compound (**I**), showing the atom-labeling scheme.

In the asymmetric unit of the title compound (**I**) (Fig. 1 ▸), the tetra­methyl­dithio­imidodiphosphinate anion is bent with a P—N—P angle of 132.16 (6)°, and chelates the Cs cation through S⋯Cs⋯N electrostatic inter­actions [S⋯Cs⋯N = 53.074 (17)°; S⋯Cs = 3.4377 (3) Å; N⋯Cs = 3.2054 (9) Å]. The bond distances of 2.0003 (4), 1.6075 (10), 1.6179 (10) and 1.9869 (4) Å for S1—P1, P1—N1, N1—P2 and P2—S2, respectively, suggest that the anion is a delocalized system (Cea-Olivares & Nöth, 1987 ▸; Churchill *et al.*, 1971 ▸). The phospho­rus atoms are in an approximately tetra­hedral environment, the average bond angles being S—P—N = 113.9°, S—P—C = 109.4°, and C—P—C = 103.4°.

## Supra­molecular features   

**Figure 2:**
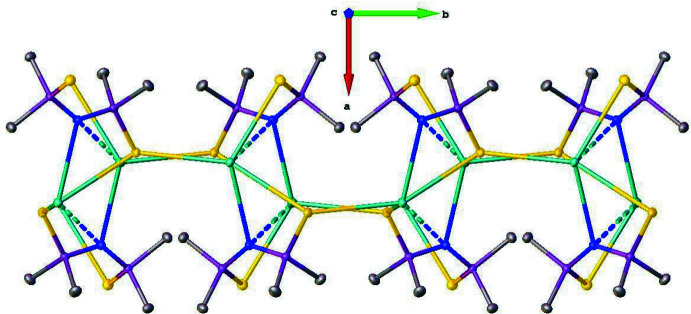
A view along the *c* axis, showing the undulating two-dimensional polymer structure. Hydrogen atoms were omitted for clarity.

**Figure 4:**
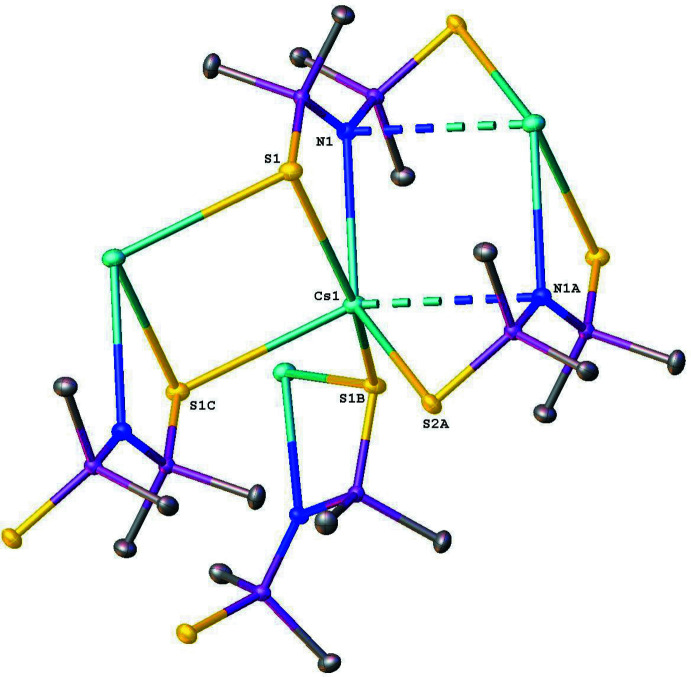
A view of the hexa­coordination of the caesium cation. Atoms with the suffix A, B or C are at the symmetry positions A: 1 − *x*, 1 − *y*, 1 − *z*; B: 1 − *x*, 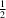 + *y*, 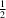 − *z*; C: 1 − *x*, 1 − *y*, −*z*. Hydrogen atoms were omitted for clarity.

In the crystal, the salt [CsMe_2_P(S)NP(S)Me_2_] (**I**) is self-assembled as an undulating supra­molecular 2D polymeric structure, which is parallel to the *bc* plane (Figs. 2 ▸ and 3 ▸). The Cs cations are hexa­coordinated and linked to four different anions by Cs⋯S and Cs⋯N electrostatic inter­actions (Fig. 4 ▸). Analysis of this CsS_4_N_2_ polyhedron with the *SHAPE 2.1* program (Llunell *et al.*, 2013 ▸) gave CShM values of 9.50434 and 8.43874 for a regular octa­hedron and a trigonal prism, respectively, meaning that the coordination environment of the cesium atom is highly irregular. These polyhedra inter­connect either by sharing vertices or an edge. The Cs⋯S ionic bond distances vary from 3.4377 (3) to 3.4726 (3) Å, which are close to the value of 3.51Å predicted from the ionic radii (Shannon, 1976 ▸). Regarding the N—Cs bond distances, two different distances were determined. One of them is 3.2054 (9) Å, which is close to the value of 3.13 Å predicted from the ionic radii, and the other is 3.651 Å, which is less than the value of 4.4 Å predicted from the van der Waals radii (Batsanov, 2001 ▸). Furthermore, five methyl groups are located in a close vicinity of the Cs^+^ cation with the Cs⋯H distance shorter than 4 Å, but only the shortest Cs1⋯H2*C*(1 – *x*, 1 – *y*, 1 – *z*) distance of 3.269 Å is similar to those observed in [LiCs(HMDS)_2_]_∞_ and can be labeled as an agostic inter­action (Ojeda-Amador *et al.*, 2016 ▸). The cyclic motifs Cs_2_S_2_, Cs_2_N_2_, Cs_2_N_2_P_2_S_2_ in this arrangement possess crystallographic inversion symmetry.

## Database survey   

The current version of the Cambridge Structural Database (Version 2021.1, updated August 2021; Groom *et al.*, 2016 ▸) contains only three cesium dichalcogenoimidodiphosphinates, (18C6)CsPh_2_P(*E*)NP(*E*)Ph_2_ (BENSAP, BENSET and BENSIX for *E* = O, S and Se; Hernández-Arganis *et al.*, 2004 ▸). Furthermore, only five compounds each containing two [Me_2_P(S)NP(S)Me_2_]^−^ ligands and one *M* ^2+^ cation (*M* = Fe, Ni, Pd, Cd, Co) are included in the database: IMSPFE10, IMSPNI10, OCANEL, TASXAN and ZACZAE (Churchill & Wormald, 1971 ▸; Churchill *et al.*, 1971 ▸; Bilic *et al.*, 2000 ▸; Ghesner *et al.*, 2005 ▸ and Silvestru *et al.*, 1995 ▸). The dinuclear species MIWYUM with two [Mn(CO)_3_]^+^ cations (Zuniga-Villarreal *et al.*, 2001 ▸) is also noteworthy. No compound with [Me_2_P(*E*)NP(*E*)Me_2_]^−^ (*E* = O or Se) is included in the database.

## Synthesis and crystallization   

Cs[Me_2_P(S)]_2_N (**I**) was obtained by the reaction of [Me_2_P(S)NHP(S)(Me_2_)] with Cs_2_CO_3_, according to a method previously described (Schmidpeter & Ebeling, 1968 ▸) and isolated solvent-free. The salt Cs[Me_2_P(S)]_2_N was recrystallized by slow evaporation from methanol. The spectroscopic data of the received sample *(vide infra)* coincided with the published ones and are therefore not reported; however, they can be consulted in the above-mentioned reference.

## Refinement   

Crystal data, data collection and structure refinement details are summarized in Table 1 ▸. H atoms were positioned geometrically (C—H = 0.98 Å) and constrained using the riding-model approximation with *U*_iso_(H) = 1.5 *U*_eq_(C).

## Supplementary Material

Crystal structure: contains datablock(s) I. DOI: 10.1107/S2056989021009798/dj2031sup1.cif

Structure factors: contains datablock(s) I. DOI: 10.1107/S2056989021009798/dj2031Isup2.hkl

CCDC reference: 2110998

Additional supporting information:  crystallographic information; 3D view; checkCIF report

## Figures and Tables

**Figure 3:**
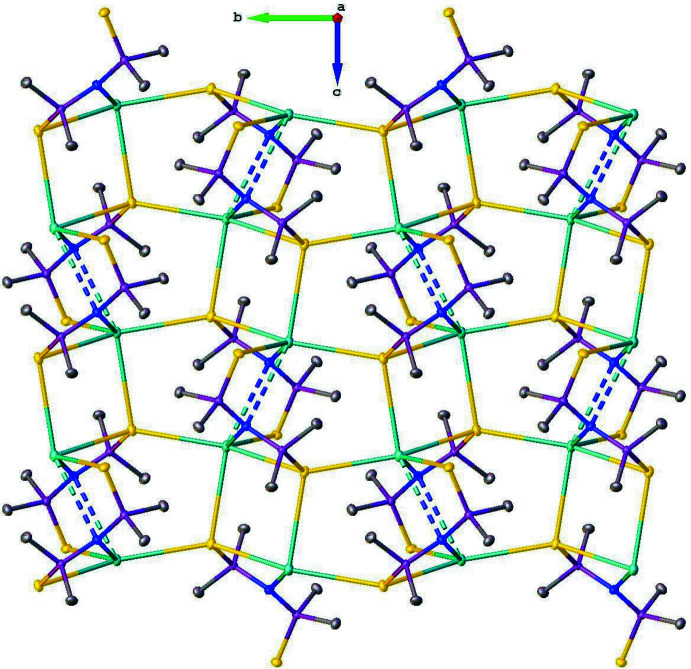
A view along the *a* axis of the supra­molecular two-dimensional polymer structure parallel to the *bc* plane. Hydrogen atoms were omitted for clarity.

**Table 1:** Experimental details

Crystal data	
Chemical formula	[Cs(C_4_H_12_NP_2_S_2_)]
*M*_r_	333.12
Crystal system, space group	Monoclinic, *P*2_1_/*c*
Temperature (K)	100
*a*, *b*, *c* (Å)	11.0320 (2), 12.4326 (3), 8.2173 (2)
β (°)	93.8752 (4)
*V* (Å^3^)	1124.48 (4)
*Z*	4
Radiation type	Mo *K*α
μ (mm^−1^)	3.89
Crystal size (mm)	0.18 × 0.15 × 0.12

Data collection	
Diffractometer	Bruker *SMART* APEXII DUO
Absorption correction	Multi-scan (*SADABS*; Bruker, 2016 ▸)
*T*_min_, *T*_max_	0.100, 0.147
No. of measured, independent and observed [*I* > 2σ(*I*)] reflections	15582, 5097, 4791
*R*_int_	0.017
(sin θ/λ)_max_ (Å^−1^)	0.833

Refinement	
*R*[*F*^2^ > 2σ(*F* ^2^)], *wR*(*F* ^2^), *S*	0.017, 0.035, 1.08
No. of reflections	5097
No. of parameters	95
H-atom treatment	H-atom parameters constrained
Δρ_max_, Δρ_min_ (e Å^−3^)	0.58, −0.76

Computer programs: *APEX3* (Bruker, 2016 ▸), *SAINT* (Bruker, 2016 ▸), *SHELXT* (Sheldrick, 2015*a* ▸), *SHELXL* (Sheldrick, 2015*b* ▸), and *OLEX2* (Dolomanov *et al.*, 2009 ▸).

## supplementary crystallographic information

### Crystal data

**Table:**

[Cs(C_4_H_12_NP_2_S_2_)]	*F*(000) = 640
*M**_r_* = 333.12	*D*_x_ = 1.968 Mg m^−^^3^
Monoclinic, *P*2_1_/*c*	Mo *K*α radiation, λ = 0.71073 Å
*a* = 11.0320 (2) Å	Cell parameters from 4791 reflections
*b* = 12.4326 (3) Å	θ = 2.4–25.2°
*c* = 8.2173 (2) Å	µ = 3.89 mm^−^^1^
β = 93.8752 (4)°	*T* = 100 K
*V* = 1124.48 (4) Å^3^	Plate, clear light white
*Z* = 4	0.18 × 0.15 × 0.12 mm

### Data collection

**Table:**

Bruker SMART APEXII DUO diffractometer	5097 independent reflections
Radiation source: Incoatec ImuS with multilayer mirrors	4791 reflections with *I* > 2σ(*I*)
Detector resolution: 8.333 pixels mm^-1^	*R*_int_ = 0.017
ω scans	θ_max_ = 36.3°, θ_min_ = 2.5°
Absorption correction: multi-scan (SADABS; Bruker, 2016)	*h* = −17→17
*T*_min_ = 0.100, *T*_max_ = 0.147	*k* = −19→20
15582 measured reflections	*l* = −13→13

### Refinement

**Table:**

Refinement on *F*^2^	Primary atom site location: dual
Least-squares matrix: full	Hydrogen site location: inferred from neighbouring sites
*R*[*F*^2^ > 2σ(*F*^2^)] = 0.017	H-atom parameters constrained
*wR*(*F*^2^) = 0.035	*w* = 1/[σ^2^(*F*_o_^2^) + (0.0129*P*)^2^ + 0.2359*P*] where *P* = (*F*_o_^2^ + 2*F*_c_^2^)/3
*S* = 1.08	(Δ/σ)_max_ = 0.004
5097 reflections	Δρ_max_ = 0.58 e Å^−^^3^
95 parameters	Δρ_min_ = −0.76 e Å^−^^3^
0 restraints	

### Special details

**Table:**

Geometry. All esds (except the esd in the dihedral angle between two l.s. planes) are estimated using the full covariance matrix. The cell esds are taken into account individually in the estimation of esds in distances, angles and torsion angles; correlations between esds in cell parameters are only used when they are defined by crystal symmetry. An approximate (isotropic) treatment of cell esds is used for estimating esds involving l.s. planes.

### Fractional atomic coordinates and isotropic or equivalent isotropic displacement parameters (Å^2^)

**Table:**

	*x*	*y*	*z*	*U*_iso_*/*U*_eq_	
Cs1	0.56629 (2)	0.59017 (2)	0.27106 (2)	0.01538 (2)	
S1	0.40180 (2)	0.36434 (2)	0.15753 (4)	0.01505 (5)	
S2	0.16545 (3)	0.55491 (2)	0.67865 (3)	0.01535 (5)	
P1	0.25673 (2)	0.42906 (2)	0.25349 (3)	0.01072 (5)	
P2	0.21826 (2)	0.61338 (2)	0.46982 (3)	0.01115 (5)	
N1	0.29485 (8)	0.53396 (8)	0.35928 (11)	0.01287 (16)	
C1	0.14280 (11)	0.45999 (10)	0.09220 (14)	0.0180 (2)	
H1A	0.1713	0.5193	0.0261	0.027*	
H1B	0.0672	0.4811	0.1397	0.027*	
H1C	0.1280	0.3964	0.0232	0.027*	
C2	0.18262 (11)	0.32802 (10)	0.37023 (15)	0.0181 (2)	
H2A	0.1574	0.2678	0.2988	0.027*	
H2B	0.1111	0.3595	0.4165	0.027*	
H2C	0.2392	0.3021	0.4586	0.027*	
C3	0.08798 (10)	0.67113 (10)	0.35577 (14)	0.0165 (2)	
H3A	0.1128	0.6988	0.2514	0.025*	
H3B	0.0551	0.7301	0.4185	0.025*	
H3C	0.0255	0.6158	0.3356	0.025*	
C4	0.31966 (11)	0.72634 (9)	0.50889 (15)	0.0169 (2)	
H4A	0.3379	0.7596	0.4052	0.025*	
H4B	0.3952	0.7012	0.5661	0.025*	
H4C	0.2807	0.7794	0.5766	0.025*	

### Atomic displacement parameters (Å^2^)

**Table:**

	*U*^11^	*U*^22^	*U*^33^	*U*^12^	*U*^13^	*U*^23^
Cs1	0.01350 (3)	0.01249 (3)	0.02027 (4)	−0.00100 (2)	0.00205 (2)	−0.00067 (2)
S1	0.01356 (11)	0.01225 (11)	0.01975 (12)	0.00045 (9)	0.00416 (9)	−0.00285 (9)
S2	0.01700 (12)	0.01704 (12)	0.01254 (11)	0.00079 (10)	0.00488 (9)	0.00192 (9)
P1	0.01031 (10)	0.01168 (11)	0.01021 (11)	−0.00022 (9)	0.00089 (8)	0.00053 (8)
P2	0.01079 (11)	0.01129 (11)	0.01158 (11)	0.00056 (9)	0.00243 (9)	0.00048 (9)
N1	0.0121 (4)	0.0131 (4)	0.0136 (4)	−0.0001 (3)	0.0023 (3)	−0.0024 (3)
C1	0.0179 (5)	0.0217 (5)	0.0138 (5)	0.0031 (4)	−0.0035 (4)	−0.0002 (4)
C2	0.0184 (5)	0.0178 (5)	0.0182 (5)	−0.0051 (4)	0.0030 (4)	0.0026 (4)
C3	0.0139 (4)	0.0177 (5)	0.0180 (5)	0.0024 (4)	0.0024 (4)	0.0046 (4)
C4	0.0167 (5)	0.0149 (5)	0.0193 (5)	−0.0021 (4)	0.0040 (4)	−0.0031 (4)

### Geometric parameters (Å, º)

**Table:**

Cs1—S1	3.4377 (3)	P1—C2	1.8090 (12)
Cs1—S1^i^	3.4726 (3)	P2—Cs1^iii^	3.9879 (3)
Cs1—S1^ii^	3.6077 (3)	P2—N1	1.6179 (10)
Cs1—S2^iii^	3.4667 (3)	P2—C3	1.8103 (11)
Cs1—N1^iii^	3.6506 (9)	P2—C4	1.8107 (12)
Cs1—N1	3.2054 (9)	N1—Cs1^iii^	3.6506 (9)
Cs1—H2A^i^	3.8388	C1—H1A	0.9800
Cs1—H2C^iii^	3.2692	C1—H1B	0.9800
Cs1—H4A	3.5176	C1—H1C	0.9800
Cs1—H4B^iv^	3.5628	C2—H2A	0.9800
Cs1—H4B	3.4588	C2—H2B	0.9800
Cs1—H4C^iv^	3.7984	C2—H2C	0.9800
S1—Cs1^v^	3.4726 (3)	C3—H3A	0.9800
S1—Cs1^ii^	3.6077 (3)	C3—H3B	0.9800
S1—P1	2.0003 (4)	C3—H3C	0.9800
S2—Cs1^iii^	3.4667 (3)	C4—H4A	0.9800
S2—P2	1.9869 (4)	C4—H4B	0.9800
P1—N1	1.6075 (10)	C4—H4C	0.9800
P1—C1	1.8052 (11)		
			
S1—Cs1—S1^i^	153.276 (4)	H4B—Cs1—H4C^iv^	69.4
S1—Cs1—S1^ii^	86.999 (7)	H4C^iv^—Cs1—H2A^i^	109.6
S1^i^—Cs1—S1^ii^	89.749 (6)	Cs1^v^—S1—Cs1^ii^	107.659 (7)
S1—Cs1—S2^iii^	92.185 (7)	Cs1—S1—Cs1^v^	135.252 (9)
S1^ii^—Cs1—N1^iii^	144.629 (15)	Cs1—S1—Cs1^ii^	93.001 (7)
S1^i^—Cs1—N1^iii^	104.024 (15)	P1—S1—Cs1^ii^	117.081 (14)
S1—Cs1—N1^iii^	93.707 (15)	P1—S1—Cs1	89.211 (12)
S1^i^—Cs1—H2A^i^	52.3	P1—S1—Cs1^v^	113.760 (14)
S1—Cs1—H2A^i^	147.1	P2—S2—Cs1^iii^	89.743 (12)
S1^ii^—Cs1—H2A^i^	68.5	S1—P1—Cs1	60.397 (11)
S1—Cs1—H4A	101.4	N1—P1—Cs1	51.35 (3)
S1^i^—Cs1—H4A	55.0	N1—P1—S1	110.64 (4)
S1—Cs1—H4B^iv^	102.3	N1—P1—C1	111.59 (5)
S1—Cs1—H4B	102.3	N1—P1—C2	112.81 (5)
S1^i^—Cs1—H4B^iv^	53.8	C1—P1—Cs1	118.41 (4)
S1^ii^—Cs1—H4C^iv^	69.8	C1—P1—S1	109.40 (4)
S1^i^—Cs1—H4C^iv^	74.0	C1—P1—C2	102.71 (6)
S1—Cs1—H4C^iv^	80.0	C2—P1—Cs1	138.82 (4)
S2^iii^—Cs1—S1^i^	114.494 (7)	C2—P1—S1	109.39 (4)
S2^iii^—Cs1—S1^ii^	93.387 (7)	S2—P2—Cs1^iii^	60.375 (11)
S2^iii^—Cs1—N1^iii^	51.243 (15)	N1—P2—Cs1^iii^	66.26 (3)
S2^iii^—Cs1—H2A^i^	68.7	N1—P2—S2	117.15 (4)
S2^iii^—Cs1—H4A	153.9	N1—P2—C3	112.20 (5)
S2^iii^—Cs1—H4B^iv^	148.8	N1—P2—C4	103.48 (5)
S2^iii^—Cs1—H4C^iv^	161.7	C3—P2—Cs1^iii^	162.13 (4)
N1—Cs1—S1	53.074 (17)	C3—P2—S2	108.87 (4)
N1—Cs1—S1^i^	105.154 (17)	C3—P2—C4	104.09 (6)
N1—Cs1—S1^ii^	114.158 (17)	C4—P2—Cs1^iii^	93.37 (4)
N1—Cs1—S2^iii^	131.503 (18)	C4—P2—S2	110.14 (4)
N1—Cs1—N1^iii^	93.76 (2)	Cs1—N1—Cs1^iii^	86.24 (2)
N1^iii^—Cs1—H2A^i^	94.3	P1—N1—Cs1^iii^	100.76 (4)
N1—Cs1—H2A^i^	157.3	P1—N1—Cs1	105.60 (4)
N1—Cs1—H2C^iii^	121.1	P1—N1—P2	132.16 (6)
N1—Cs1—H4A	50.2	P2—N1—Cs1	121.68 (4)
N1—Cs1—H4B^iv^	78.0	P2—N1—Cs1^iii^	89.80 (4)
N1—Cs1—H4B	50.7	P1—C1—H1A	109.5
N1^iii^—Cs1—H4C^iv^	145.1	P1—C1—H1B	109.5
N1—Cs1—H4C^iv^	55.1	P1—C1—H1C	109.5
H2C^iii^—Cs1—S1^i^	55.1	H1A—C1—H1B	109.5
H2C^iii^—Cs1—S1^ii^	119.8	H1A—C1—H1C	109.5
H2C^iii^—Cs1—S1	146.0	H1B—C1—H1C	109.5
H2C^iii^—Cs1—S2^iii^	67.4	P1—C2—H2A	109.5
H2C^iii^—Cs1—N1^iii^	52.3	P1—C2—H2B	109.5
H2C^iii^—Cs1—H2A^i^	51.3	P1—C2—H2C	109.5
H2C^iii^—Cs1—H4A	89.6	H2A—C2—H2B	109.5
H2C^iii^—Cs1—H4B	74.0	H2A—C2—H2C	109.5
H2C^iii^—Cs1—H4B^iv^	109.0	H2B—C2—H2C	109.5
H2C^iii^—Cs1—H4C^iv^	126.8	P2—C3—H3A	109.5
H4A—Cs1—S1^ii^	109.3	P2—C3—H3B	109.5
H4A—Cs1—N1^iii^	105.2	P2—C3—H3C	109.5
H4A—Cs1—H2A^i^	107.2	H3A—C3—H3B	109.5
H4A—Cs1—H4B^iv^	49.1	H3A—C3—H3C	109.5
H4A—Cs1—H4C^iv^	44.4	H3B—C3—H3C	109.5
H4B—Cs1—S1^ii^	135.7	Cs1—C4—H4A	63.3
H4B—Cs1—S1^i^	62.8	Cs1—C4—H4B	59.7
H4B^iv^—Cs1—S1^ii^	60.5	Cs1—C4—H4C	159.4
H4B—Cs1—S2^iii^	128.8	P2—C4—Cs1	91.05 (4)
H4B—Cs1—N1^iii^	78.7	P2—C4—H4A	109.5
H4B^iv^—Cs1—N1^iii^	151.7	P2—C4—H4B	109.5
H4B—Cs1—H2A^i^	110.6	P2—C4—H4C	109.5
H4B^iv^—Cs1—H2A^i^	84.9	H4A—C4—H4B	109.5
H4B—Cs1—H4A	26.5	H4A—C4—H4C	109.5
H4B—Cs1—H4B^iv^	75.2	H4B—C4—H4C	109.5
H4B^iv^—Cs1—H4C^iv^	24.8		

Symmetry codes: (i) −*x*+1, *y*+1/2, −*z*+1/2; (ii) −*x*+1, −*y*+1, −*z*; (iii) −*x*+1, −*y*+1, −*z*+1; (iv) *x*, −*y*+3/2, *z*−1/2; (v) −*x*+1, *y*−1/2, −*z*+1/2.

## References

[bb1] Batsanov, S. S. (2001). *Inorg. Mat.* **37**, 871–885.

[bb2] Bilic, D., Silvestru, A., Silvestru, C., Haiduc, I. & Drake, J. E. (2000). *Rev. Soc. Quim. Mex.* **44**, 116–121.

[bb29] Bruker (2016). *APEX2*, *SAINT* and *SADABS*. Bruker AXS Inc., Madison, Wisconsin, USA.

[bb3] Cea-Olivares, R. & Muñoz, M. A. (1993). *Monats. Chem.* **124**, 471–476.

[bb4] Cea-Olivares, R. & Nöth, H. (1987). *Z. Naturforsch. Teil B*, **42**, 1507–1509.

[bb5] Churchill, M. R., Cooke, J., Fennessey, J. P. & Wormald, J. (1971). *Inorg. Chem.* **10**, 1031–1035.

[bb6] Churchill, M. R. & Wormald, J. (1971). *Inorg. Chem.* **10**, 1778–1782.

[bb7] Dolomanov, O. V., Bourhis, L. J., Gildea, R. J., Howard, J. A. K. & Puschmann, H. (2009). *J. Appl. Cryst.* **42**, 339–341.

[bb8] Ghesner, M., Silvestru, A., Silvestru, C., Drake, J. E., Hursthouse, M. B. & Light, M. E. (2005). *Inorg. Chim. Acta*, **358**, 3724–3734.

[bb9] Groom, C. R., Bruno, I. J., Lightfoot, M. P. & Ward, S. C. (2016). *Acta Cryst.* B**72**, 171–179. 10.1107/S2052520616003954 PMC4822653 27048719

[bb10] Haiduc, I. & Silaghi-Dumitrescu, I. (1986). *Coord. Chem. Rev.* **74**, 127–270.

[bb11] Hernández-Arganis, M., Hernández-Ortega, S., Toscano, R. A., García-Montalvo, V. & Cea-Olivares, R. (2004). *Chem. Commun.* pp. 310–311. 10.1039/b311524k 14740052

[bb12] Leung, W. H., Zheng, H., Chim, J. L. C., Chan, J., Wong, W. T. & Williams, I. D. (2000). *J. Chem. Soc. Dalton Trans.* pp. 423–430.

[bb13] Llunell, M., Casanova, D., Cirera, J., Alemany, P. & Alvarez, S. (2013). *SHAPE. Program for the Stereochemical Analysis of Molecular Fragments by Means of Continuous Shape Measures and Associated Tools.* Universitat de Barcelona, Barcelona.

[bb14] Ma, X. F., Luo, X. F., Yan, Z. P., Wu, Z. G., Zhao, Y., Zheng, Y. X. & Zuo, J. L. (2019). *Organometallics*, **38**, 3553–3559.

[bb15] Ojeda-Amador, A. I., Martínez-Martínez, A. J., Kennedy, A. R. & O’Hara, C. T. (2016). *Inorg. Chem.* **55**, 5719–5728. 10.1021/acs.inorgchem.6b00839 27177080

[bb16] Preez, J. G. H. du, Knabl, K. U., Krüger, L. & van Brecht, B. J. A. M. (1992). *Solvent Extr. Ion Exch.* **10**, 729–748.

[bb17] Rudler, H., Denise, B. & Gregorio, J. R. (1997). *Chem. Commun.* pp. 2299–2300.

[bb18] Schmidpeter, A. & Ebeling, J. (1968). *Chem. Ber.* **101**, 815–823.

[bb19] Shannon, R. D. (1976). *Acta Cryst.* A**32**, 751–767.

[bb21] Sheldrick, G. M. (2015*a*). *Acta Cryst.* A**71**, 3–8.

[bb22] Sheldrick, G. M. (2015*b*). *Acta Cryst.* C**71**, 3–8.

[bb23] Silvestru, C., Roesler, R., Haiduc, I., Cea-Olivares, R. & Espinosa-Perez, G. (1995). *Inorg. Chem.* **34**, 3352–3354.

[bb24] Silvestru, S. & Drake, J. E. (2001). *Coord. Chem. Rev.* **223**, 117–216.

[bb25] Slawin, A. M. Z., Ward, J., Williams, D. J. & Woollins, J. D. (1994). *J. Chem. Soc. Chem. Commun.* pp. 421–422.

[bb26] Woollins, J. D. (1996). *J. Chem. Soc. Dalton Trans.* pp. 2893–2901.

[bb27] Yamazaki, Y., Tsukuda, T., Furukawa, S., Dairiki, A., Sawamura, S. & Tsubomura, T. (2020). *Inorg. Chem.* **59**, 12375–12384. 10.1021/acs.inorgchem.0c01445 32830956

[bb28] Zúñiga-Villarreal, N., Reyes-Lezama, M. & Espinosa, G. (2001). *J. Organomet. Chem.* **626**, 113–117.

